# Supplementary material for: Evidence accumulation relates to perceptual consciousness and monitoring
Source: Nat Commun. 2021 May 31;12:3261. doi: 10.1038/s41467-021-23540-y (PMC8166835; doi:10.1038/s41467-021-23540-y)
Supplement: Supplementary file 1 — Supplementary Information [file 41467_2021_23540_MOESM1_ESM.pdf]

Evidence accumulation relates to perceptual consciousness and monitoring

Supplementary Notes

Experiments 1 - 2

We verified that stimulus intensity did not vary significantly between hits and misses during Experiment 1 (t(272)=0.52, p=0.60) nor during Experiment 2 (t(101)=1.36, p=0.18). In Experiment 2, a response was considered correct when the participant answered “yes” following a stimulus (hit) or “no” in the absence of a stimulus (correct rejection): hits and correct rejections represented respectively 39.69% and 20.61% of trials. Incorrect trials included “no” responses following a stimulus (miss) and “yes” responses in the absence of a stimulus (false alarms), which represented respectively 38.93% and 0.80%. This corresponded to a d-prime of 1.64 and a bias of 1.64 indicative of conservative behavior (i.e., tendency to answer no in the presence of a stimulus) and consistent with healthy controls described below. Importantly, stimulus onset did not vary significantly between hits and misses (t(101)=-0.59, p=0.56). Confidence was analyzed separately for “yes” and “no” responses.

Experiment 4

A response was considered correct when participants answered “yes” following a stimulus (hit) or “no” in the absence of a stimulus (correct rejection): hits and correct rejections represented respectively 37.81%±1.70 and 18.9%±0.66 of trials. Incorrect trials included “no” responses following a stimulus (miss) and “yes” responses in the absence of a stimulus (false alarms), which represented respectively 42.07%±1.67 and 1.37%±0.71 of trials. This corresponded to an average d-prime of 1.67±0.26, and a bias of 7.62±3.53 indicative of conservative behavior (i.e., tendency to answer no in the presence of a stimulus). Importantly, stimulus intensity and stimulus onset did not vary significantly between hits and misses (t(17)=0.81, p=0.43 and t(17)=0.12, p=0.91 , respectively). Confidence was analyzed separately for “yes” and “no” responses. For “yes” responses, average confidence was higher in hits (77.79%±3.82) than in false alarms (62.16%±7.15; t(23.12)=-3.78, p=0.001). For “no” responses, confidence was higher in correct rejections (86.31%±3.47) than in misses (83.06%±3.44; t(17)=5.59, p<0.001). These results indicate higher confidence for correct than incorrect responses (confidence gap: t(32)=4.10, p<0.001), implying that participants had a metacognitive access to tactile detection processes.

Choice probabilities (Experiment 2)

We confirmed the factorial analysis reported in the main text by probing whether the specificity for detection correlated with that of confidence using neuronal choice probabilities (CP). Choice probability was computed using the area under the curve (AUC) for detection and normalized to the [-1,1] interval: CP = 2*AUC-1. We found that choice probabilities in the population of neurons was positively related to the correlation between spike counts and confidence in hits (R = 0.54, p = 0.001; Supplementary Fig. 4a), suggesting that evidence for detection led to higher confidence. On the contrary, choice probability was not related to the correlation between spike counts and confidence in misses (R = -0.04, p = 0.71; Supplementary Fig. 4b), neither was the correlation between spike counts and confidence in hits related to the correlation between spike counts and confidence in misses (R = -0.06, p = 0.75; Supplementary Fig. 4c).

Analysis of confidence variance (Experiment 4)

To further support our evidence accumulation model, we verified some of its predictions a-posteriori. Notably, our model predicted lower variance for simulated confidence in misses (0.012±0.001) than in hits (0.026±0.002; z=3.2, p=0.0012), since accumulated evidence was bounded between zero and the

decision bound for misses but not for hits. Similarly, in the data, confidence ratings had lower variance for misses (0.015±0.002) compared to hits (0.026±0.002; z=2.6, p=0.0084). Furthermore, since the drift rate was null for correct rejections, the model predicted even lower variance of confidence for correct rejections (0.007±0.001) compared to misses (z=3.7; p=0.002), which we confirmed in the data: (0.011±0.001 vs. 0.015±0.002; z=3.1; p=0.0018).

Supplementary figures

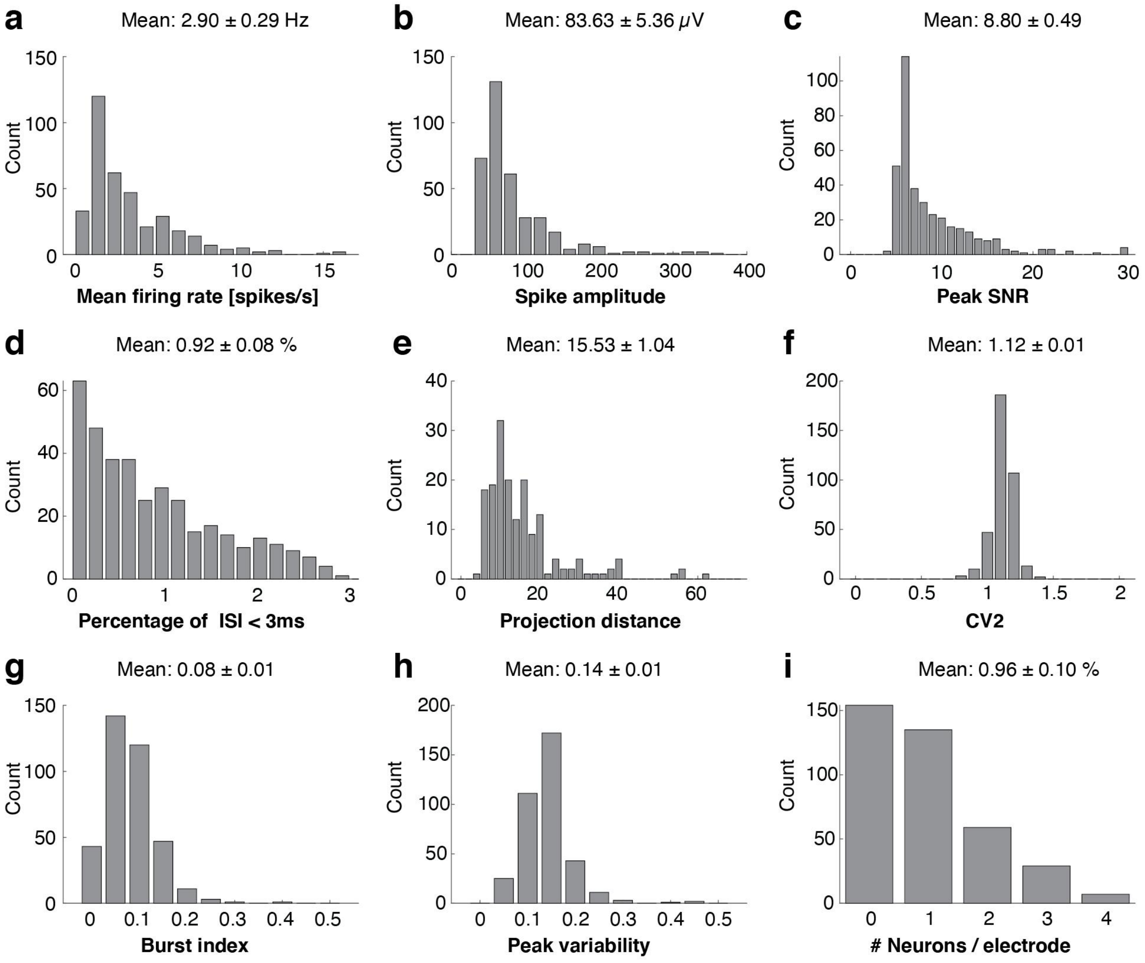

Supplementary Fig. 1. *Spike sorting statistics.* ***(a)*** *Histogram of firing rates.* ***(b)*** *Histogram of mean spike amplitudes (at the peak, rectified, since all neurons had negative action potentials).* ***(c)*** *Histogram of signal-to-noise ratio (ratio between spike peak amplitude and estimated noise).* ***(d)*** *Histogram of the percentage of inter-spike interval lower than a 3 ms refractory period.* ***(e)*** *Histogram of projection distance (Rutishauser et al., 2006) for electrodes with more than one single unit.* ***(f)*** *Histogram of modified coefficient of variation.* ***(g)*** *Histogram of burst index (number of inter-spike intervals lower than 10 ms).* ***(h)*** *Histogram of peak variability (ratio between the standard deviation of spike amplitude and the mean spike amplitude).* ***(i)*** *Histogram of number of neurons found per electrode.*

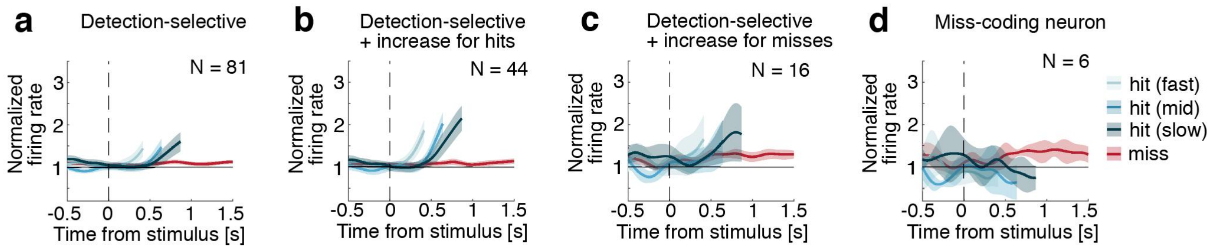

Supplementary Fig 2. *Average firing rates of different types of responsive neurons in Experiment 1. Firing rates were normalized using a 0.3 s pre-stimulus baseline, for three bins of RT (hits; blue) and for misses (red). (**a**) Detection-selective neurons, (**b**) Subset of **a** with an increase in firing rate compared to baseline. (**c**) Subset of **a** with an increase in firing rate for misses but not as high as for hits. (**d**) Subset of **a** with an increase in firing rate for misses which was stronger than for hits. All shaded areas represent 95%-CI.*

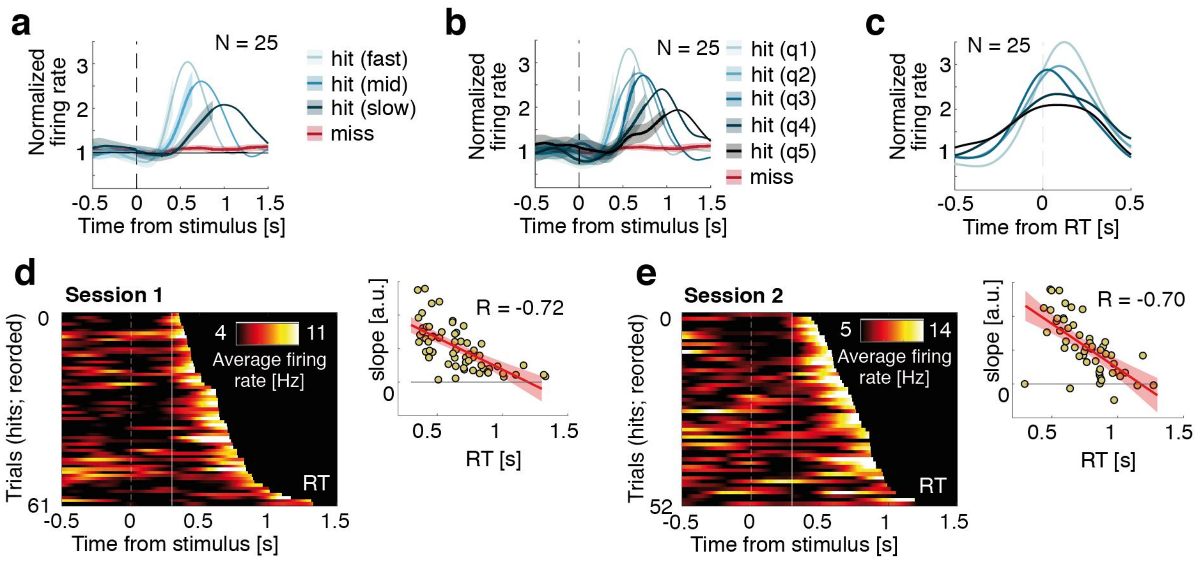

Supplementary Fig 3. *Average firing rates of detection- and RT-selective single- and multi-units, combined (Experiment 1). Firing rates are normalized using a 0.3 s pre-stimulus baseline, for three bins of RT (hits; blue) and for misses (red). (**a**) Aligned to stimulus onset for three bins of RT (hits; blue) and for misses (red). (**b**) Aligned to stimulus onset for four bins of RT (hits; blue) and for misses (red). Traces without shaded areas represent firing rates after the median RT for each bin and suggest that evidence accumulation continues after the decision. (**c**) Firing rates for the four RT bins of **b**, aligned to the response time (RT). (**d,e**) Firing rates for single trials, averaged across the 25 detection- and RT-selective single- and multi-units, reordered by RT for the two sessions. Insets represent the slope of the firing rates between 300 ms and the RT, as a function of the RT. All shaded areas represent 95%-CI. Of note, this is the only figure in this study including multi-unit data.*

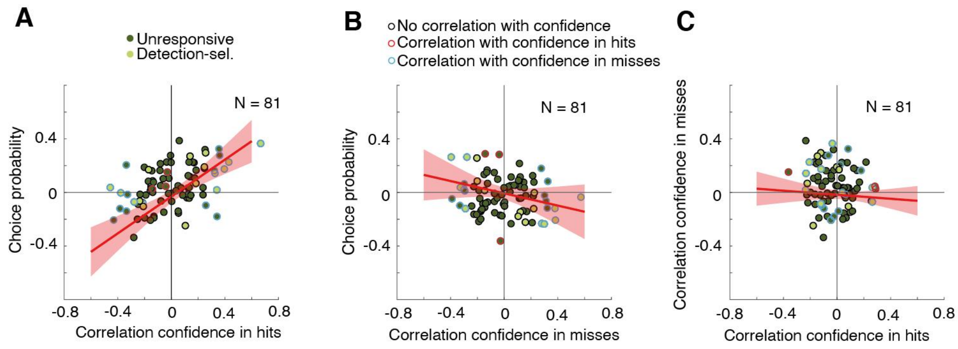

Supplementary Fig 4. *Choice probability and correlation analysis. Each panel depicts individual neurons, as a function of different measures: (a) Correlation between confidence in hits and spike rate (x-axis) and choice probability (y-axis). (b) Correlation between confidence in hits and spike rate (x-axis) and choice probability (y-axis). (c) Correlation between confidence in hits and spike rate (x-axis) and correlation between confidence in misses and spike rate (y-axis). Regression lines are represented in red with shaded areas representing 95%-CI. Unresponsive neurons are in dark green and responsive neurons in light green (neurons with an interaction effect between detection and confidence). Neurons with a number of spikes correlating with confidence in hits (respectively misses) are circled in cyan (resp. red).*

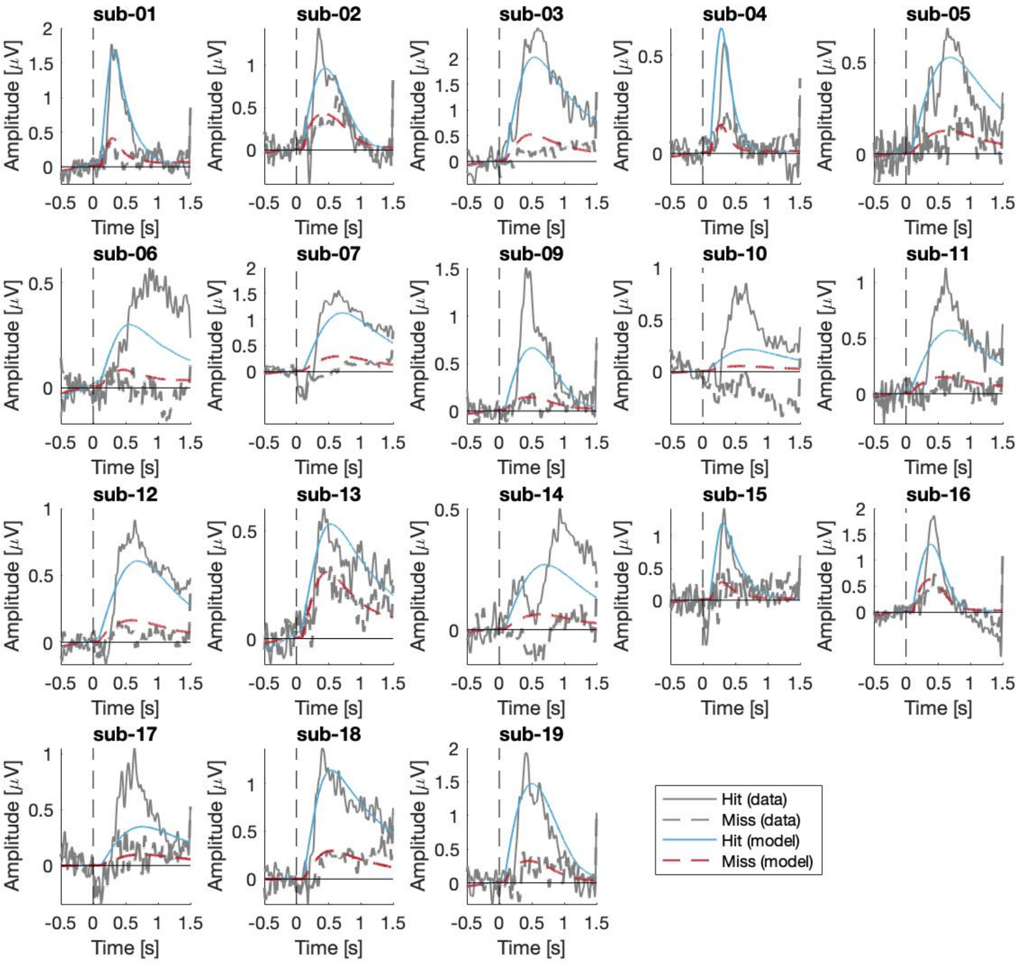

Supplementary Fig 5. *Model fits in Experiment 4. Each plot represents the individual fit of EEG data for hits (cyan traces) and misses (red traces). Observed data for hits (upper line) and misses (lower line) are shown in grey. Vertical dashed line represents stimulus onset. Average R across participants: 0.70±0.03.*

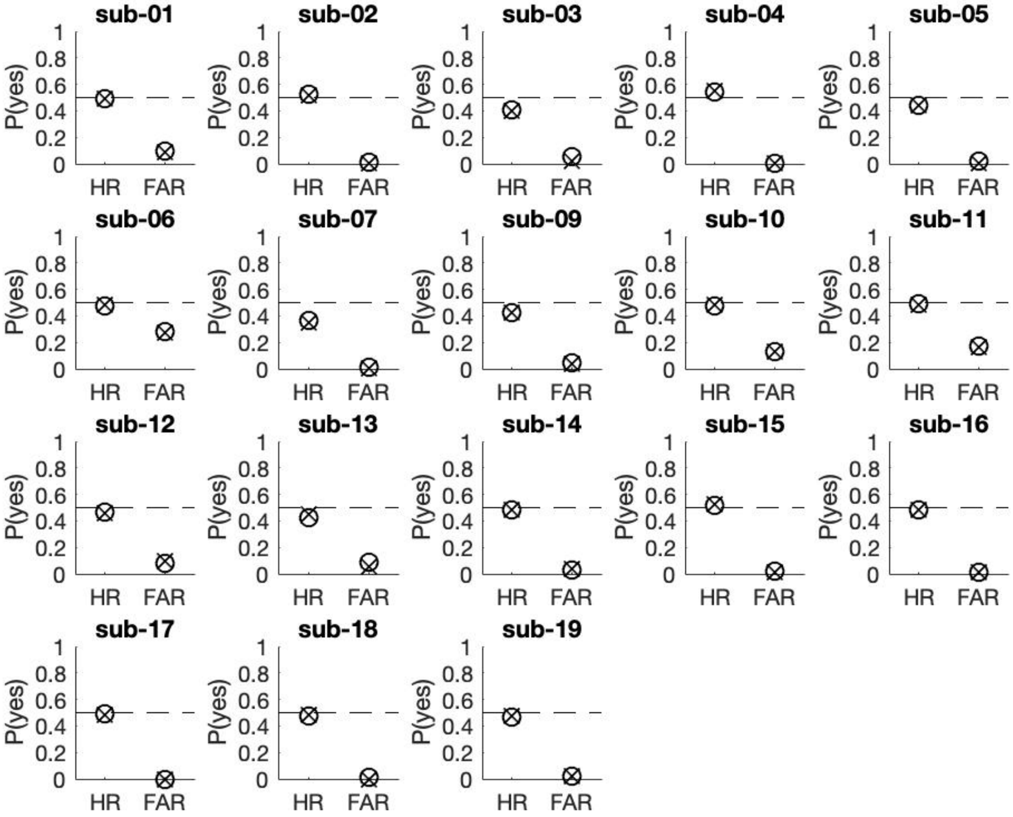

Supplementary Fig 6. *Model fits in Experiment 4. Each plot represents individual fits of detection reports, summarized as hit rate (HR) and false alarm rate (FAR). Circles show data and crosses show model simulations. Horizontal dashed shows the 50% hit rate target. Correlation: R between participants: 0.96 for hit rate and 0.95 for false alarm rate.*

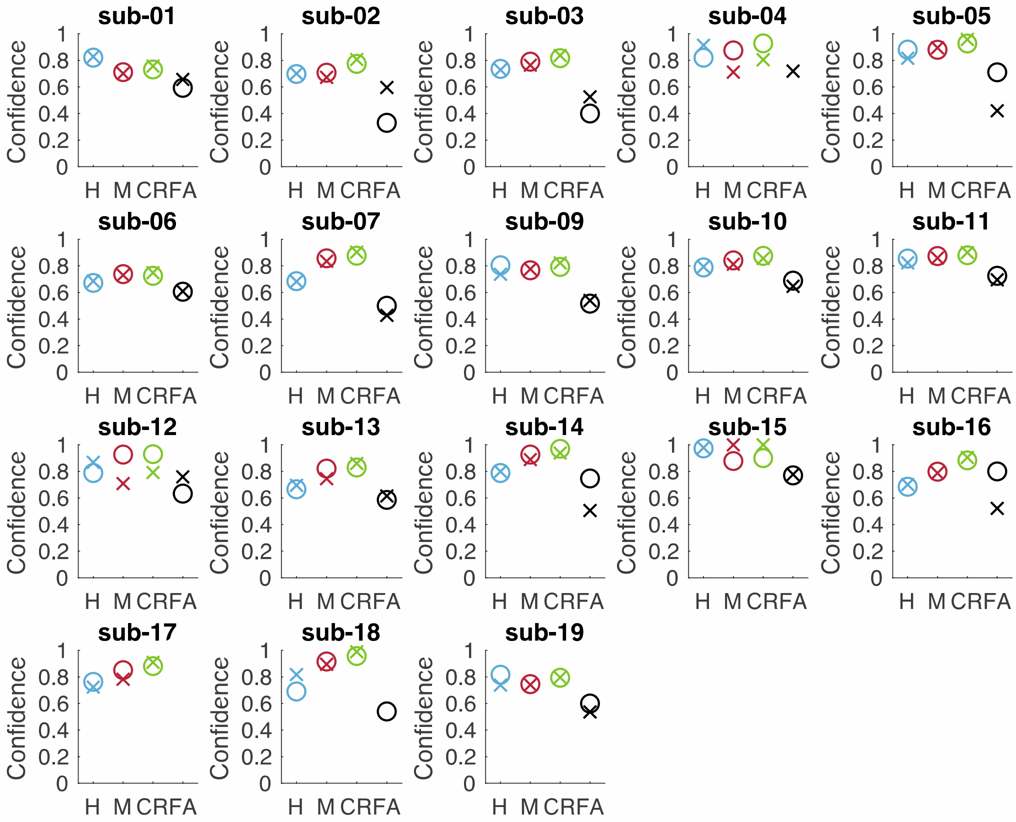

Supplementary Fig 7. *Model fits in Experiment 4. Each plot represents individual fits of confidence ratings for hits (H), misses (M), correct rejections (CR) and false alarms (FA; when any). Circles show data and crosses show model simulations. Average R across participants 0.83±0.03 for hits, 0.85±0.03 for misses, 0.81±0.04 for correct rejections and 0.45±0.09 for false alarms.*

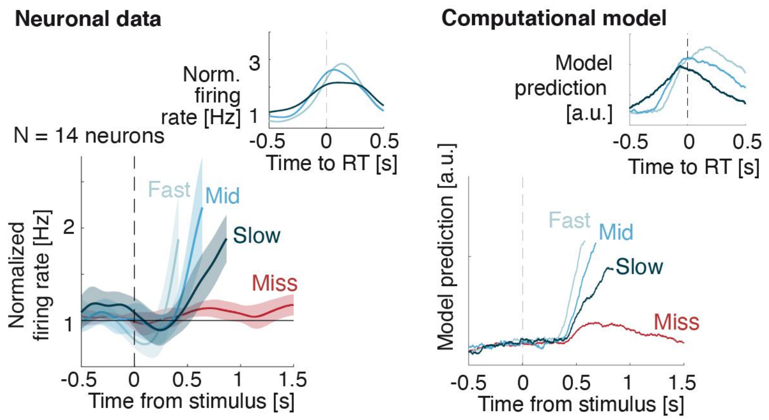

Supplementary Fig 8. *Comparison between neuronal data and evidence accumulation traces simulated by the computational model. Left: Average firing rates for detection- and RT- responsive neurons, for three bins of RT (hits; blue) and for misses (red). Firing rates were normalized to a 0.3 s pre-stimulus baseline and time-locked to the stimulus onset. Inset: Corresponding average firing rates time-locked to the response. The data are identical to Fig. 1F, displayed here for comparison purposes. Right: Average evidence accumulation traces, time-locked to the stimulus onset, as simulated by the model for a set of manually selected parameters. Inset: Corresponding average evidence accumulation traces time-locked to the response time of the model. To make the simulation more realistic, we separated the non-decision time into a perceptual component (corresponding to the delay for perceptual evidence to start being accumulated) and a motor component, corresponding to the delay between the decision and the motor response. We set the motor delay to 80 ms (Resulaj et al., 2009, Nat. Neuro) and added a small amount of variability to both components (Gaussian random noise with a standard deviation of 40 ms). a.u.: arbitrary units.*
