# Supplementary material for: Evidence accumulation relates to perceptual consciousness and monitoring
Source: Nat Commun. 2021 May 31;12:3261. doi: 10.1038/s41467-021-23540-y (PMC8166835; doi:10.1038/s41467-021-23540-y)
Supplement: Supplementary file 3 — Reporting Summary [file 41467_2021_23540_MOESM3_ESM.pdf]

nature research

Corresponding author(s): Nathan Faivre

Last updated by author(s): Apr 29, 2021

Reporting Summary

Nature Research wishes to improve the reproducibility of the work that we publish. This form provides structure for consistency and transparency in reporting. For further information on Nature Research policies, see our Editorial Policies and the Editorial Policy Checklist.

Statistics

For all statistical analyses, confirm that the following items are present in the figure legend, table legend, main text, or Methods section.

n/a	Confirmed	
☐	☒	The exact sample size (*n*) for each experimental group/condition, given as a discrete number and unit of measurement
☐	☒	A statement on whether measurements were taken from distinct samples or whether the same sample was measured repeatedly
☐	☒	The statistical test(s) used AND whether they are one- or two-sided<br>*Only common tests should be described solely by name; describe more complex techniques in the Methods section.*
☐	☒	A description of all covariates tested
☐	☒	A description of any assumptions or corrections, such as tests of normality and adjustment for multiple comparisons
☐	☒	A full description of the statistical parameters including central tendency (e.g. means) or other basic estimates (e.g. regression coefficient) AND variation (e.g. standard deviation) or associated estimates of uncertainty (e.g. confidence intervals)
☐	☒	For null hypothesis testing, the test statistic (e.g. *F*, *t*, *r*) with confidence intervals, effect sizes, degrees of freedom and *P* value noted<br>*Give P values as exact values whenever suitable.*
☒	☐	For Bayesian analysis, information on the choice of priors and Markov chain Monte Carlo settings
☒	☐	For hierarchical and complex designs, identification of the appropriate level for tests and full reporting of outcomes
☐	☒	Estimates of effect sizes (e.g. Cohen's *d*, Pearson's *r*), indicating how they were calculated

Our web collection on statistics for biologists contains articles on many of the points above.

Software and code

Policy information about availability of computer code

Data collection	Behavior: Custom code written with Matlab v9.4.0.813654 (R2018a) and Psychtoolbox v3.0.16. Microelectrode: Neuroport recording system; ECoG: Brain Quick LTM; EEG: WaveGuard EEG; Available on https://doi.org/10.18112/openneuro.ds001785.v1.1.1
Data analysis	Custom code written with Matlab v9.4.0.813654 (R2018a) using Osort v4.10 and EEGLAB 2019.1.0 with SASICA v1.3.4, iELVis (Aug 18, 2020 git commit). Available on https://doi.org/10.17605/OSF.IO/YHXDB

For manuscripts utilizing custom algorithms or software that are central to the research but not yet described in published literature, software must be made available to editors and reviewers. We strongly encourage code deposition in a community repository (e.g. GitHub). See the Nature Research guidelines for submitting code & software for further information.

Data

Policy information about availability of data

All manuscripts must include a data availability statement. This statement should provide the following information, where applicable:

- Accession codes, unique identifiers, or web links for publicly available datasets
- A list of figures that have associated raw data
- A description of any restrictions on data availability

Behavioral, electrocorticographic and electroencephalographic data that support the findings of this study have been deposited in the OpenNeuro repository (https://doi.org/10.18112/openneuro.ds001785.v1.1.1). Microelectrode data are available upon written request, dependent upon the establishment of a data sharing agreement between the trial's investigator, sponsor, and interested third party.

Field-specific reporting

Please select the one below that is the best fit for your research. If you are not sure, read the appropriate sections before making your selection.

☒ Life sciences ☐ Behavioural & social sciences ☐ Ecological, evolutionary & environmental sciences

For a reference copy of the document with all sections, see nature.com/documents/nr-reporting-summary-flat.pdf

Life sciences study design

All studies must disclose on these points even when the disclosure is negative.

Sample size	Experiments 1, 2 and 3 are based on a single participant with a rare implant, and no sample size calculation was performed. For Experiment 4, we recruited 20 participants, similar to our previous work using similar recordings (Faivre et al., 2018; no sample size calculation was performed).
Data exclusions	We excluded trials with epileptic activity as it could corrupt our signal of interest. We excluded two participants in Experiment 4 due to artifacts in the data that prevented us from measuring clean evoked responses. In both cases, criteria were defined after data collection and were not predermined.
Replication	We replicated the single-neuron findings with ECoG recordings and EEG on 18 healthy participants. These two attempts at replication were successful.
Randomization	Participants were not grouped and hence no randomization was performed. Trial order was fully randomized for each subject.
Blinding	Data collection was performed automatically and experimenters were blind to the experimental conditions (occurence of catch trials). No blinding was involved during data analysis, as we relied on a fully automatized procedure that did no require data manipulation.

Reporting for specific materials, systems and methods

We require information from authors about some types of materials, experimental systems and methods used in many studies. Here, indicate whether each material, system or method listed is relevant to your study. If you are not sure if a list item applies to your research, read the appropriate section before selecting a response.

Materials & experimental systems

n/a	Involved in the study	
☒	☐	Antibodies
☒	☐	Eukaryotic cell lines
☒	☐	Palaeontology and archaeology
☒	☐	Animals and other organisms
☐	☒	Human research participants
☒	☐	Clinical data
☒	☐	Dual use research of concern

Methods

n/a	Involved in the study	
☒	☐	ChIP-seq
☒	☐	Flow cytometry
☐	☒	MRI-based neuroimaging

Human research participants

Policy information about studies involving human research participants

Population characteristics	One participant was a young human patient suffering from drug-resistant epilepsy due to a focal cortical dysplasia in the left central sulcus. We also recruited 18 healthy participants (7 females; age: 25.2 years, SD = 4.1)
Recruitment	The participant with epilepsy was recruited during invasive epilepsy monitoring. The 18 healthy participants were students that took part in this study in exchange for monetary compensation. No systematic recruitment bias were expected to alter the results.
Ethics oversight	The present study was approved by the Commission Cantonale d'Ethique de la Recherche de la République et Canton de Genève (2016-01856) and (2015-00092 15-273).

Note that full information on the approval of the study protocol must also be provided in the manuscript.

Magnetic resonance imaging

Experimental design

Design type	Anatomical scan for clinical purposes
Design specifications	Anatomical scan for clinical purposes
Behavioral performance measures	n/a

Acquisition

Imaging type(s)	Anatomical
Field strength	3T
Sequence & imaging parameters	Structural T1-weighted images (TR = 5000, TE = 2.88, flip angle: 0°), with 1 x 1 x 1mm voxels (FOV = 256 mm)
Area of acquisition	whole brain

Diffusion MRI ☐ Used ☒ Not used

Preprocessing

Preprocessing software	FreeSurfer
Normalization	n/a
Normalization template	n/a
Noise and artifact removal	n/a
Volume censoring	n/a

Statistical modeling & inference

Model type and settings	n/a
Effect(s) tested	n/a

Specify type of analysis: ☒ Whole brain ☐ ROI-based ☐ Both

Statistic type for inference (See Eklund et al. 2016)	n/a
Correction	n/a

Models & analysis

n/a	Involved in the study
☒	☐ Functional and/or effective connectivity
☒	☐ Graph analysis
☒	☐ Multivariate modeling or predictive analysis
